# Detection of chromosomal instability using ultrasensitive chromosomal aneuploidy detection in the diagnosis of precancerous lesions of gastric cancer

**DOI:** 10.3389/fgene.2024.1359231

**Published:** 2024-04-09

**Authors:** Suting Qian, Feifei Xie, Haoyu Zhao, Ting Jiang, Yi Sang, Wei Ye, Qingsheng Liu, Danli Cai

**Affiliations:** ^1^ Hangzhou Hospital of TCM Affiliated to Zhejiang Chinese Medical University, Hangzhou, China; ^2^ Intensive Care Unit, The First Affiliated hospital of Zhejiang Chinese Medical University (Zhejiang Provincial Hospital of Chinese Medicine), Hangzhou, China

**Keywords:** ultrasensitive chromosomal aneuploidy detection, chromosome instability, copy number variations, precancerous lesions of gastric cancer, gene

## Abstract

**Background:**

The diagnosis of Precancerous Lesions of Gastric Cancer (PLGC) is challenging in clinical practice. We conducted a clinical study by analyzing the information of relevant chromosome copy number variations (CNV) in the TCGA database followed by the UCAD technique to evaluate the value of Chromosomal Instability (CIN) assay in the diagnosis of PLGC.

**Methods:**

Based on the screening of gastric cancer related data in TCGA database, CNV analysis was performed to explore the information of chromosome CNV related to gastric cancer. Based on the gastroscopic pathology results, 12 specimens of patients with severe atrophy were screened to analyze the paraffin specimens of gastric mucosa by UCAD technology, and to explore the influence of related factors on them.

**Results:**

The results of CNV in TCGA database suggested that chromosome 7, 8, and 17 amplification was obvious in patients with gastric cancer. UCAD results confirmed that in 12 patients with pathologic diagnosis of severe atrophy, five of them had positive results of CIN, with a positive detection rate of 41.7%, which was mainly manifested in chromosome seven and chromosome eight segments amplification. We also found that intestinalization and HP infection were less associated with CIN. And the sensitivity of CIN measurement results was significantly better than that of tumor indicators.

**Conclusion:**

The findings suggest that the diagnosis of PLGC can be aided by UCAD detection of CIN, of which Chr7 and 8 may be closely related to PLGC.

## 1 Introduction

Gastric cancer is one of the most common malignant tumors of the gastrointestinal tract and has the fifth highest incidence rate worldwide. The incidence of gastric cancer has declined in most countries as a result of advances in current diagnostic and treatment options, but the mortality rate due to gastric cancer remains among the highest in the world (Sun et al., 2022). Currently, the diagnosis of gastric cancer relies on serum tumor markers, pepsinogen, gastroscopy and pathological histology. However, the lack of clear indications due to the lack of obvious symptoms in the early stages of gastric cancer has led to many people being diagnosed only in the late stages of gastric cancer, thus limiting treatment options (Qiu et al., 2021).

Carcinoembryonic antigen CEA, CA 19-9 and CA 72-4 are the most commonly used serological tumor markers for the diagnosis of gastric cancer (Feng et al., 2017; Rosu et al., 2023). Some studies have shown that elevated levels of CEA, CA19-9 and CA 72–4 have low sensitivity and specificity for the early diagnosis of gastric cancer, but are strongly associated with poor prognosis (Feng et al., 2017; Xu et al., 2021). Elevated CA19-9 is also common in patients with pancreatitis and pancreatic cancer (Fahrmann et al., 2021). Pepsin is an aspartic acid protease secreted by gastric mucosal cells, which mainly consists of proteasinogen I and pepsinogen II (Miki, 1999). Pepsinogen can reflect the function and state of the gastric mucosa and is closely related to gastric lesions (Han et al., 2022). When the PgI/PgII ratio decreases, it is usually considered to be the best serological marker for gastric mucosal atrophy, which further suggests precancerous gastric lesions and can prevent gastric cancer to a certain extent, but the diagnosis of early gastric cancer still lacks specificity (Leja and Linē, 2021; Malfertheiner et al., 2017).

Gastroscopy combined with histopathological examination has definite diagnostic significance in screening for early gastric cancer. However, most tissue-based biomarkers for gastric cancer are at risk of assumption error, thereby increasing the rate of missed diagnoses due to tumor heterogeneity.

In recent years, a biomarker assay for the analysis of cancer cells or cancer cell derivatives, known as a liquid biopsy, has been progressively developed (Febbo et al., 2020). Liquid biopsies are primarily used for early cancer detection, disease progression and prognosis (Siravegna et al., 2017). The most common analytes for liquid biopsies are circulating tumor cells and circulating free nucleic acids. Recent studies have demonstrated that the detection of circulating tumor cells may have potential use in the early detection of gastric cancer, particularly in relation to information on circulating tumor DNA fragment length, DNA copy number variation, etc (Campos-Carrillo et al., 2020).

Chromosome instability (CIN) is a persistent abnormal chromosome segregation in cancer cells compared to normal cells, which is mainly reflected by abnormal somatic copy number, accompanied by focal amplification of oncogenes or deletion of tumor suppressor genes (Bach et al., 2019). CIN is one of the major forms of genomic instability in a wide range of human cancers, and it is present in most solid malignancies, as well as being central to the evolution of cancer (Bakhoum and Cantley, 2018; Turajlic et al., 2020). CIN as a new alternative diagnostic tool and driver of tumorigenesis. It has been shown that CIN influences tumorigenesis and progression, including driving intra-tumor heterogeneity, leading to spatial and temporal diversification of tumor subclones, facilitating metastasis, accelerating tumor phenotypic adaptation, achieving cellular immortality, escaping immune surveillance, and developing resistance to drug therapy (Sansregret et al., 2018).

The Cancer Genome Atlas (TCGA) covers all genomic features of human cancers. We borrowed information from this database to study the effect of CIN on gastric cancer, analyzed the chromosome copy number changes in the database, affirmed the features of associated CIN caused by gastric cancer, and linked the chromosomal features associated with gastric cancer to clinical information.

In this experiment, with the help of Ultrasensitive Chromosomal Aneuploidy Detection (UCAD) technology, the gene tissue DNA extracted from gastric mucosal tissue was used as a specimen to detect chromosomal instability in the subject at the genome-wide level by applying Low Coverage Whole Genome Sequencing (LC-WGS) with bioinformatics analysis to achieve qualitative and quantitative detection of chromosomal stability. This method is of guiding significance in assisting early cancer diagnosis, late prevention of cancer progression, early intervention and prognostic assessment. UCAD analysis of CIN provides a new and unexplored field for determining and predicting the severity and prognosis of patients with PLGC. Therefore, we conducted this study with the aim of evaluating whether CIN has a diagnostic role and prognostic value in identifying gastric precancerous lesions.

## 2 Materials and methods

### 2.1 CNV analysis of the TCGA database TCGA

Data from TCGA database were used in this study. A total of 181,797 data of TCGA chromosome copy number related to gastric cancer were downloaded from TCGA, and 1,595,984 data were obtained by selecting the latest version of “SNP6 GRCh38 Remapped Probeset File for Copy Number Variation Analysis” file from Genepattern. Genes in CNV regions were annotated using Genome Research Consortium Human build 38 (GRCh38) as the reference genome. The data were sorted out and analyzed online using Gistic 2.0 with a confidence level of 0.90, and the data were screened to obtain the visual analysis using the R package “maftools”, which yielded information about the correlation between the relevant chromosomes and gastric cancer.

### 2.2 Patients’ characteristics and ethics statement

We collected 45 patients with suspected gastric mucosal atrophy/PLGC who underwent gastroscopy at our endoscopy centre between January 2020 and January 2022. We excluded 15 patients who did not want to take part in the study and 18 patients with mild to moderate atrophy confirmed by pathological biopsy. Finally, a total of 12 patients with severe atrophy could be analyzed.

**FIGURE 1:**
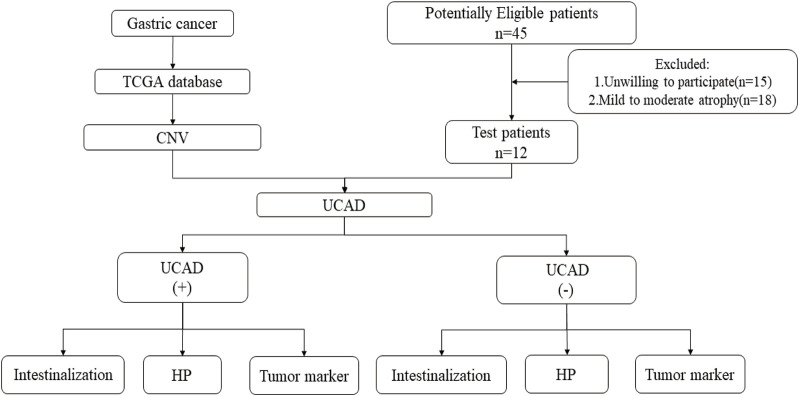
Research flowchart.

Formalin Fixation and Paraffin Embedding (FFPE) samples were collected from 12 PLGC patients to take part in the UCAD test. The design and methods of the study with human subjects were comprehensively described in the study protocol. The study has been approved by the ethical review committees of Hangzhou Hospital of Traditional Chinese Medicine. Informed consent was obtained from all patients. The study procedure is shown in Figure 1.

Inclusion and exclusion criteria:

Inclusion Criteria:

(1) Patients with chronic atrophic gastritis;
(2) Gastroscopic biopsy indicated severe intestinal metaplasia;

Exclusion criteria:

(1) patients with Subtotal gastrectomy;
(2) Patients who have been diagnosed with stomach cancer;
(3) Patients with other malignant tumors;
  All participants underwent standard collection of blood samples to examine tumor markers. We focused on CA 19-9、CEA、CA 724examinations as recommended by the NCCN guideline. The cutoff values for tumor biomarkers were: CA19-9, 37.0 U/mL; CEA, 5 ng/mL; CA7-24, IU/m.

### 2.3 UCAD and LC-WGS test

DNA is isolated from gastric mucosal tissue by column extraction using Magen Kit, and three methods were used for nucleic acid quality control. The degree of DNA degradation and RNA contamination were analyzed by agarose gel electrophoresis; the purity of DNA is measured by a nanodrop (OD260/280 ratio); Qubit can accurately measure the concentration of DNA; Whole genome DNA qualified was fragmented into small fraction by using Physical method or zymochemistry method. Library construction is about adding splices to small sequencing fragments. DNA fragments are subjected to end repair, which repairs the DNA fragments to flat ends; splices are added; and the U-joints are converted to Y-joints; magnetic beads are purified and impurities are removed; and PCR amplification is performed, with the addition of INDEX and two types of oligonucleotides that are complementary to the sequencer chip. The second magnetic bead purification, the removal of impurities such as polymerase again, and finally quality testing, including DNA concentration testing, agarose gel electrophoresis, and fragment length testing, are required to complete the library construction.

DNA replication using library fragments as templates for bridge amplification and single base extension sequencing.

### 2.4 Statistical analysis

Gastric mucosal tissue DNA was extracted and analyzed by Illumina X10. At least 10 million paired reads were collected for each sample. Circular binary segmentation algorithm from R package, DNA Copy, was then used to detect significant genomic breakpoints and copy number-changed genomic segments. R package “DNACopy” was used to analyze copy-number changes. *p* = 0.05 was considered as statistically significant binary segmentation. Absolute segment value was used for further analysis. A *p*-value <0.05 was considered as statistically significant. For categorical variables, the χ2 test was used as appropriate. All statistical analyses were performed using SPSS22.0. Proportion trend tests were used to analyze the associations between clinicopathologic UCAD screening positivity and clinicopathologic parameters. Data are reported as means and SDs, medians and interquartile ranges, and HRs or ORs with 95% confidence intervals (CIs), as appropriate. All analyses were performed with the use of R software, version 3.4.3.

## 3 Results

### 3.1 Differences in chromosomal variants associated with gastric cancer in the TCGA database

**FIGURE 2:**
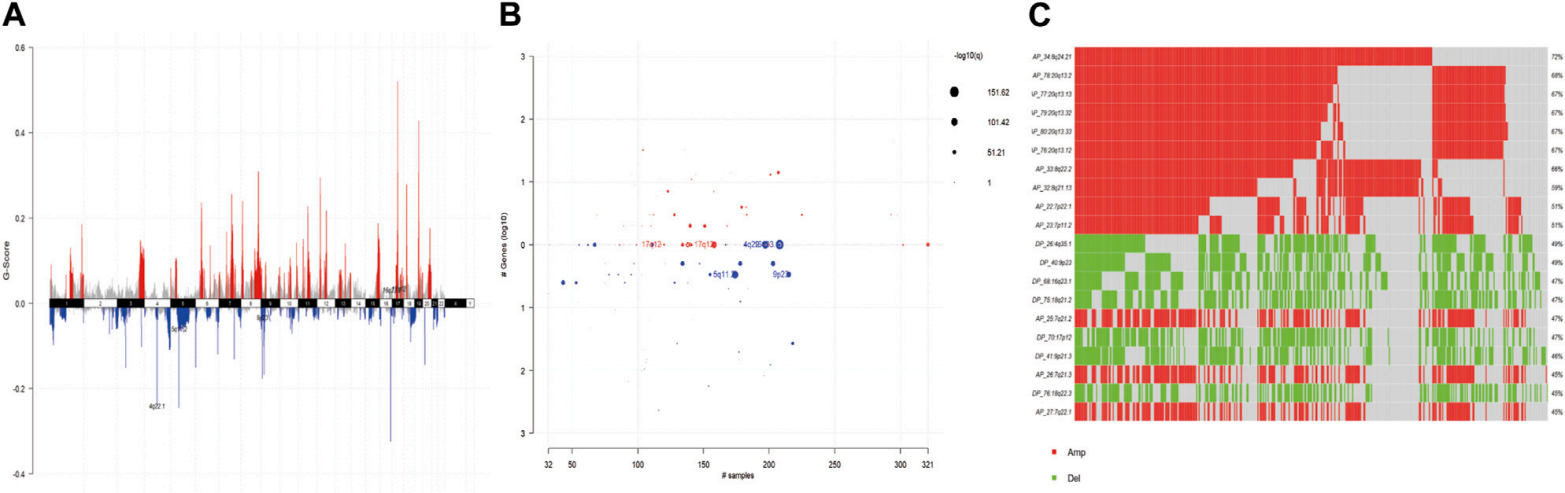
**(A)** G-scores assigned by GISTIC2.0 for every cytoband plotted along the chromosome. **(B)** GISTIC2.0 results plotted as function of altered cytobands, mutated samples, and genes involved within the cytoband. Size of each bubble is according to -log10 transformed q values. **(C)** Oncoplot displays most frequently altered (amplifications or deletions) copy number events ordered according to the frequency. Each columns represents a sample and each row represent a CNV segment.

For the copy number variation data in the TCGA database, we used GISTIC 2.0 to identify genes with significant amplification or deletion, and the results showed that CNV amplification was significant in gastric cancer patients, with significant amplification on chromosomes 7, 8, and 17, and significant deletion on chromosomes 4, 5, and 9, with more than 50% of chromosomal amplification on chr-8, chr-20, and chr-7 (8q24.21, 20q13.2, 20q13.13, 20q13.32, 20q13.33, 20q13.12, 8q22.2, 8q21.13, 7q22.1, 7q11.2). The copy number variation distribution is shown in Figures 2A–C.

### 3.2 Patient characterization analysis

A total of 12 patients with severe atrophy could be analyzed. The median age was 57 years and eight patients (66.7%) were male. The baseline characteristics of these patients were shown in Table 1. Details was provided in Supplementary Table S1.

**TABLE 1:** The baseline characteristics of patients.

	Patients
Sex	
male	8
female	4
Age	
≥57years	6
<57years	6
History of alcohol and tobacco	
smoke	4
drink	3
Dietary habit	
Heavy	4
Light	8
Basic diseases	
Have	7
Not	5

### 3.3 CIN profiles

**FIGURE 3:**
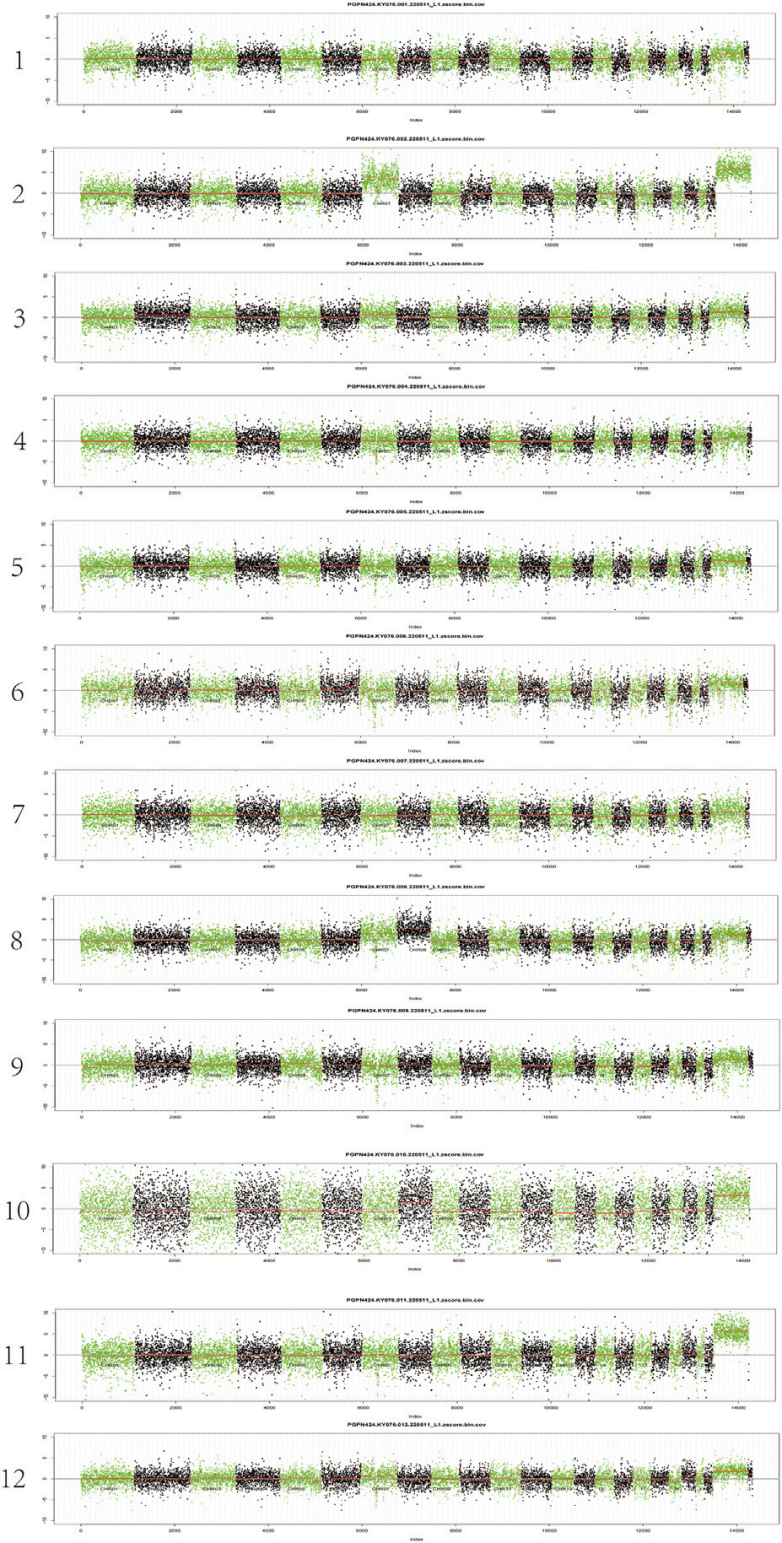
The chromosome profiles of patients.

The positive rate of CIN was 41.7% (5/12) in patients with severe atrophy. The chromosome profiles of all patients were shown in Figure 3. As shown by the test results, the genomic aberrations in patients with severe atrophy may be found on chr-7 and chr-8, mainly manifested as amplification of chr-7 and chr-8.

### 3.4 Correlation of UCAD with intestinalization

As shown in Table 2, 11 of 12 (91.6%) samples were accompanied by intestinalization, of which 5/11 (45.45%) showed mutations in chr7p+/chr8p+(*p* > 0.05). Intestinalization typically occurs in association with severe atrophy. Nearly half of the intestinalization samples exhibited mutations in chr7p+/chr8p+.

**TABLE 2:** Correlation of UCAD with intestinalization.

	Intestinalization	
	+	-
UCAD chr7p+/8p+	5	0
UCAD Neg	6	1
P	*p* > *0.05*	

### 3.5 Correlation of UCAD with HP

As shown in Table 3, five of 12 (41.67%) samples were accompanied with HP infection, of which 1/5 (20%) showed mutations in chr7p+/chr8p+(*p* > 0.05). Greater than 40% of the severe atrophies were accompanied by an HP infection. Additionally, the proportion of mutations in chr7p+/chr8p+ was higher in non-HP-infected samples.

**TABLE 3:** Correlation of UCAD with HP.

	HP	
	+	-
UCAD chr7p+/8p+	1	4
UCAD Neg	4	3
P	*p* > *0.05*	

### 3.6 Correlation of UCAD with tumor marker

Serum tumor markers were tested, including AFP, CEA, CA125, CA199, CA242, CA153, CA50, CA724, SCC and FER. As shown in Table 4 of 12 (33.3%) samples were were associated with elevated tumor markers, of which 1/4 (25%) showed mutations in chr7p+/chr8p+(*p* > 0.05). The proportion of mutations in chr7p+/chr8p+ was higher in specimens negative for tumor markers than in positive samples.

**TABLE 4:** Correlation of UCAD with tumor marker.

	tumor marker	
	+	-
UCAD chr7p+/8p+	1	4
UCAD Neg	3	4
P	*p* > *0.05*	

## 4 Discussion

The etiology of GC is complex and variable. Genetic factors, epigenetic factors, and the many related genes and chromosomes they regulate can lead to complex heterogeneity of tumors, which poses major difficulties in clinical diagnosis and personalized treatment (Machlowska et al., 2020). Therefore, it is particularly important to find effective tests during gastric precancerous lesions. The continuous development of high-throughput sequencing technology has made it possible to conduct comprehensive, multilevel studies of tumors at the genomic and transcriptomic levels (Liu et al., 2023). On the basis of the existing available multi-omics data, combined with the patient’s clinical information for comprehensive research, it is more conducive to identifying effective therapeutic targets and prognostic indicators for the disease.

CIN is a phenotype in which cancer cells show either CNA or Structural Chromosomal Instability (S-CIN) compared to normal cells (Bach et al., 2019). CIN is considered to be the most fundamental cause of cancer development and is present in almost all malignant tumors (Hanahan and Weinberg, 2011). It occurs when cancer cells undergo uneven distribution of chromosomes in the daughter cells during mitosis and this chromosome segregation process continues to be erroneous, leading to changes in chromosome copy number or amplification or deletion of internal segments of chromosomes (Kneissig et al., 2019). CIN is a driver of tumorigenesis, which means that once chromosomal instability has occurred in certain parts of the patient’s body, cancer has occurred or is about to occur (Venkatesan et al., 2021). Studies have shown that CIN affects tumorigenesis and progression, including a series of processes that drive intra-tumor heterogeneity, lead to spatiotemporal diversification of tumor subclones, promote tumor metastasis, accelerate tumor phenotypic adaptation, achieve cellular immortality, escape immune surveillance, and escape drug therapy to form drug resistance (Sansregret et al., 2018).

We statistically analyzed the effect of relevant factors on chromosomal instability. Enterosis is one of the histopathological preneoplastic lesions of the stomach and is considered an important predisposing factor for the development of intestinal-type GC, for which there is no specific treatment, and regular monitoring and prevention of IM in high-risk patients is one of the main therapeutic approaches recommended by the guidelines (Jonaitis et al., 2021). Our study found that severe atrophy is often associated with intestinalization, but intestinalization is less associated with CIN positivity. *Helicobacter pylori* (HP) infection is widely recognized as an essential cause of non-cardia gastric cancer, accounting for nearly 90% of non-cardia gastric cancer cases. Previous studies have shown that one of the etiologic factors for gastric cardia cancer is H. pylori-associated atrophic gastritis, similar to noncardia cancer (Yan et al., 2022). However, probably because of the small sample size, a positive association between HP positivity and CIN positivity was also not found in this study. Tumor indicators are often used clinically as predictors of tumors, and among the available tumor markers, carcinoembryonic antigen (CEA), carbohydrate antigen CA 19-9, and CA72-4 are widely used in the follow-up of patients with gastrointestinal malignancies. These markers have been shown to be helpful in the diagnosis, treatment and prognosis of gastric cancer (Chen et al., 2017). However, we found that fewer tumor indicators were elevated in patients with severe atrophy, which could not suggest the extent of gastric precancerous lesions in patients at that stage. Therefore, we consider that the UCAD technique can be applied clinically as a predictor of gastric precancerous lesions.

In this study, we analyzed the copy number variation and mutation data based on TCGA database, from which we screened the information of relevant chromosomal mutations, we found that patients with gastric cancer were mostly seen to have obvious chr-7+, chr-8+ amplification, so we collected clinical data using UCAD technology to verify the results, the results showed that the features of chromosomal abnormality mapping in patients with severe atrophy were mainly manifested in chr-7q+ and chr-8q+ of chromosome segments. Among them, chromosome seven amplification leads to high expression of epidermal growth factor EGFR (proto-oncogene), and chromosome eight amplification leads to high expression of proto-oncogene MYC, which promotes tumor progression, suggesting that these patients are at a higher risk of cancer development.

In various cancer models, chromosome seven is often reported to contain regions that undergo genetic changes or regions of instability. However, since most of the previously mentioned studies have used either the mid-chromosome or the BAC array CGH, there has been no systematic search for individual genes undergoing copy number gain or amplification. Human chromosome seven is approximately 159 Mb in length and contains 1,150 genes and 940″pseudogenes,” many of which have been implicated in a variety of human diseases, including cystic fibrosis, deafness, B-cell lymphomas, and cancers. Chromosome seven contains known oncogenes that exhibit gene amplification, including the epidermal growth factor receptor (EGFR, located at 7p12), hepatocyte growth factor (HGF, 7q21.1), and met proto-oncogene (met/HGFR, 7q31). Since one of the most common mechanisms of oncogenic activation is gene amplification, it is crucial to be able to identify the full set of possible genes that are amplified in the tumor tissue of a given cancer model. Among them is EGFR, the expression product of the proto-oncogene HER-1, a key oncogene in gastric cancer, whose receptor tyrosine kinase activity triggers key signaling pathways for tumor cell growth and survival (Fusco et al., 2013).

Chromosome eight is a moderately long autosomal unit in humans with an extremely high mutation rate by positive selection (Nusbaum et al., 2006). If its telomeres are shortened it may be a mechanism that promotes the development of chromosomal instability during aging and chronic disease. This relatively high genomic instability of chromosome eight is found not only in evolution, but also in a variety of mutant diseases such as tumorigenesis and further invasion/metastasis. Amplification of chromosome 8q is strongly associated with intestinal-type gastric cancer (Chia and Tan, 2016). One study comparative genomic hybridization (CGH) assessed DNA copy number aberrations (CNAs) in 53 tumors, combining them with clinicopathological features and status of TP53, with 8q abnormalities accounting for 43% (Wu et al., 2001). A study using large-scale label-free proteomic quantification identified 8p21-p23 defects in the development of digestive organ tumors (Zhang et al., 2013). It has been demonstrated that chromosome eight amplification leads to high expression of the proto-oncogene MYC. c-Myc has the ability to regulate the development of many types of human cancer tumors by orchestrating gene expression, and its aberrant expression is a key driver of colorectal cancer progression (Sun et al., 2020). One of the c-Myc genes is localized to chromosome 8q24.two to three and encodes a nuclear transcription factor that regulates cell proliferation, differentiation and apoptosis (Obara et al., 2001). Karyotyping and phenotyping of circulating tumor cells (CTCs) revealed that in advanced gastric cancer (AGC) patients, different CTCs with varying chromosome eight ploidies correlated with either sensitivity or resistance to chemotherapeutic drugs (Li et al., 2014). Therefore, Amplification of chromosome 8 may promote tumor development.

Our findings suggest that chr7p+, chr8p + may be able to serve as an independent predictor of cancer. We compared the score of UCAD with that of enterochemistry, HP infection and conventional cancer tumor biomarkers. In comparison, the score of UCAD was significantly higher than that of conventional prediction. UCAD may be another noninvasive biomarker for cancer prediction. Although the results of our data are informative, due to the small sample size, a large prospective clinical trial is needed to further confirm the reliability of the results.

## Data Availability

Publicly available datasets were analyzed in this study. This data can be found here: TCGA (https://tcga-data.nci.nih.gov/tcga/).
