# Supplementary material for: Detection of chromosomal instability using ultrasensitive chromosomal aneuploidy detection in the diagnosis of precancerous lesions of gastric cancer
Source: Front Genet. 2024 Apr 9;15:1359231. doi: 10.3389/fgene.2024.1359231 (PMC11040259; doi:10.3389/fgene.2024.1359231)
Supplement: Supplementary file 1 [file Table1.docx]

Supplementary Material

# Supplementary Table S

| Number | Gender | Age | Pathological Diagnosis | Tumor Marker | Drinking and Smoking History | HP | CIN |
| --- | --- | --- | --- | --- | --- | --- | --- |
| 1 | male | 56 | severe atrophy | SCC | D&S | - | - |
| 2 | female | 62 | severe atrophy/ severe intestinalization | / | / | - | - |
| 3 | male | 54 | severe atrophy/ severe intestinalization | / | S | - | 8+ |
| 4 | female | 35 | severe atrophy/ severe intestinalization | / | / | + | - |
| 5 | male | 50 | severe atrophy/ severe intestinalization/low-grade intraepithelial neoplasia (LGIN) | CA242 | D&S | + | 7+ |
| 6 | male | 61 | severe atrophy/ severe intestinalization | / | S | + | - |
| 7 | male | 52 | severe atrophy/ severe intestinalization | CA724 | / | + | - |
| 8 | male | 62 | severe atrophy/ severe intestinalization | / | / | - | 7+8+ |
| 9 | male | 58 | severe atrophy/ severe intestinalization | / | / | - | 7+ |
| 10 | female | 42 | severe atrophy/ severe intestinalization | FER | / | + | - |
| 11 | female | 61 | severe atrophy/ severe intestinalization | / | / | - | 7+ |
| 12 | male | 66 | severe atrophy/ severe intestinalization | / | D | - | - |

Supplementary Table S Details of 12 patients with severe atrophy
